# Semantic Segmentation of Digestive Abnormalities from WCE Images by Using AttResU-Net Architecture

**DOI:** 10.3390/life13030719

**Published:** 2023-03-07

**Authors:** Samira Lafraxo, Meryem Souaidi, Mohamed El Ansari, Lahcen Koutti

**Affiliations:** 1 LabSIV, Department of Computer Science, Faculty of Sciences, Ibn Zohr University, Agadir 80000, Morocco; 2 Informatics and Applications Laboratory, Department of Computer Science, Faculty of Sciences, Moulay Ismail University, Meknes 52000, Morocco

**Keywords:** gastrointestinal tract, segmentation, deep learning, U-Net, WCE, colonoscopy, attention mechanism, residual block

## Abstract

Colorectal cancer is one of the most common malignancies and the leading cause of cancer death worldwide. Wireless capsule endoscopy is currently the most frequent method for detecting precancerous digestive diseases. Thus, precise and early polyps segmentation has significant clinical value in reducing the probability of cancer development. However, the manual examination is a time-consuming and tedious task for doctors. Therefore, scientists have proposed many computational techniques to automatically segment the anomalies from endoscopic images. In this paper, we present an end-to-end 2D attention residual U-Net architecture (AttResU-Net), which concurrently integrates the attention mechanism and residual units into U-Net for further polyp and bleeding segmentation performance enhancement. To reduce outside areas in an input image while emphasizing salient features, AttResU-Net inserts a sequence of attention units among related downsampling and upsampling steps. On the other hand, the residual block propagates information across layers, allowing for the construction of a deeper neural network capable of solving the vanishing gradient issue in each encoder. This improves the channel interdependencies while lowering the computational cost. Multiple publicly available datasets were employed in this work, to evaluate and verify the proposed method. Our highest-performing model was AttResU-Net, on the MICCAI 2017 WCE dataset, which achieved an accuracy of 99.16%, a Dice coefficient of 94.91%, and a Jaccard index of 90.32%. The experiment findings show that the proposed AttResU-Net overcomes its baselines and provides performance comparable to existing polyp segmentation approaches.

## 1. Introduction

The gastrointestinal (GI) tract is the tubular duct that connects the mouth to the anus. It is a long, continuous tube that runs from the mouth to the anus, and is responsible for the process of digestion. It is divided into two main regions: the upper GI tract (including the mouth, pharynx, esophagus, stomach) and the lower GI tract (including the small intestine, large intestine, rectum, and anus). The GI tract is lined with a mucous membrane and muscular layers that work together to move food through the tract and break it down for absorption and elimination [1].

The GI tract is also vulnerable to various medical disorders that may necessitate examination by medical specialists. Gastrointestinal illnesses, tissue inflammations, and irregular growth are examples of these. Acid reflux, for example, can induce changes in the lining of the esophagus, an unusual response can create inflammations, and proliferate cells can cluster together to form a polyp on the colon lining. These anomalies, like ulcers or sores, can be dangerous themselves. Another potential is that some anomalies can emerge, such as polyps that might become cancerous. Adenomatous polyps are polyps that have the potential to become malignant over time. Colorectal cancer (CRC) frequently begins as a precancerous polyp that can be benign or malignant. If left untreated, it is predicted that 30% of adenomas may progress to malignancy. Polyp cancer incidence varies based on the kind of polyp and population, although it is usually regarded as a common disease. According to the American Cancer Society, approximately one in every 20 persons may acquire colorectal cancer from an adenoma over their lifetime. Polyp screening, particularly for adenomatous polyps, is an essential method of preventing colorectal cancer.

Colonoscopy, which allows the doctor to see inside the rectum and colon, is currently the most effective screening method for detecting precancerous polyps and early stage colorectal cancer. Colorectal cancer (CRC) is the world’s third-most prevalent disease in males and the second-most common cancer in women. In 2018, there were approximately 1.8 million new cases and 0.9 million deaths from CRC, according to the World Health Organization (WHO). It is also a frequent disease in the United States, with an expected 148,810 new cases and 53,200 deaths in 2021. It is more frequent in older persons, with around 90% of occurrences occurring in those over the age of 50. However, due to increased screening, early identification, and improved treatment, the incidence of colorectal cancer is decreasing in many industrialized nations. Hence, visual evaluation of organs in the GI system is required by medical practitioners [1]. The most common types of stomach issues are gastrointestinal (GI) anomalies such as bleeding, pylorus, erosion, ulcers, and polyps, which require considerable medical treatment because stomach abnormalities induce a variety of diseases. In 2018, stomach cancer was one of the top five most common cancers globally, according to the World Health Organization (WHO) report.

Endoscopy traditionally gives an interior view of the GI tract. Furthermore, because the small intestine is so extensive and convoluted, this old approach cannot view it. Furthermore, patients find this endoscopic approach unpleasant and uncomfortable. Wireless capsule endoscopy is a medical procedure that allows users to see inside the gastrointestinal tract. It involves ingesting a small, pill-sized capsule that contains a camera and wireless transmitting device. The capsule takes about 60,000 images and transmits them to a receiver worn by the patient as it travels through the digestive system. The images are then used by a doctor to diagnose and monitor conditions such as Crohn’s disease, ulcerative colitis, and other digestive tract disorders. The procedure is noninvasive, safe, and does not require sedation, making it a popular alternative to traditional endoscopic procedures [2].

The primary diseases diagnosed by the WCE include ulcers, bleeding, pylorus, erosion, and polyps in the digestive tract. The WCE images are utilized to identify anatomical landmarks, pathological abnormalities, and polyp removal, all of which are important GI illnesses. Providing a range of images makes it easier to identify tumors and gastrointestinal hemorrhages, especially in the small intestine, which is now more properly studied [3]. It took a long time to analyze every image taken from each patient [4]. Furthermore, there is occasionally a high resemblance rate in distinct contextual images; this is why most expert doctors confront obstacles that require a significant amount of time to examine all of the data. This commonly leads to misdiagnosis due to clinician ineptitude or carelessness [5]. All of these challenges motivate researchers to develop a computer-aided bleeding-detection approach in order to reduce the pressure on gastroenterologists. With its ever-expanding uses, certain approaches for detecting bleeding images in WCE video have been developed [6,7].

Medical image segmentation is the process of distinguishing pixels from medical images that contain lesions. It gives details regarding the sizes and forms of areas of interest (ROIs) [8,9,10]. Previously, automatic segmentation approaches were based on both, traditional image processing and handcrafted features [11]. Deep learning techniques have recently made substantial advances in medical imaging [12]. These techniques based on their structure may be classified into two categories. Some models are based on the encoder–decoder structure like U-Net, FCN, and SegNet. Although others can maintain appropriate spatial information by employing fewer downsample operations and dilated convolution (Deep Lab-V3) [13].

In this study, we propose the AttResU-Net architecture for gastrointestinal image segmentation. We tested our model by using three publicly available datasets. Our experimental results show that the enhanced model is efficient and outperforms the popular U-Net and V-Net designs. In summary, the following are the paper’s contributions.

(1) We present the AttResU-Net architecture, a semantic segmentation neural network that uses residual blocks and attention blocks. When compared to other cutting-edge approaches, AttResU-Net markedly enhanced segmentation findings for colorectal bleeding and polyps. Even with fewer images, the suggested architecture works effectively.
(2) We test the improved architecture on a diverse collection of datasets, including MICCAI 2017, Kvasir-SEG, and CVC-ClinicDB, and compare our suggested techniques to current state-of-the-art algorithms. Furthermore, we compared our findings to those of other recent studies.
(3) Our approach detects tiny, flat, or sessile polyps that are typically overlooked during colonoscopy, which is a significant advantage over previous studies.

The remainder of this paper is organized as follows. Section 2 examines a few interesting works. Section 3 is devoted to the description of architecture. Baseline experiments are detailed in Section 4. Finally, Section 5 brings the paper to a close and offers future directions.

## 2. Literature Review

During the last few decades, many efforts have been undertaken by researchers to develop computer-aided frameworks to segment and detect gastrointestinal lesions [12,14,15,16]. Polyp segmentation has specifically been given consideration. The efficiency of the AI algorithms has approached the degree of expertise of experienced endoscopists.

Prior works were particularly centered around handcrafted descriptor-based feature learning. Handcrafted features that are focused on shape, texture, edges, and color are extracted and fed to classifiers that isolate lesions from the background. Hwang and Celebi [17] suggested an unsupervised approach for detecting polyps in VCE. Watershed segmentation was proposed, along with an initial marker selection technique based on Gabor texture features and K-means clustering. Curvature-based geometric information was used to extract polyp pixels from the obtained segments. Fu et al. [18] take into consideration arbitrary form automatically picked by superpixel segmentation. In this technique, the red ratio in RGB space is used to define the attributes of each superpixel, which are then aggregated to train a binary classifier. Ganz et al. [19] and Mamonov et al. [20] utilize shape and contour information for polyps segmentation. Color and texture information was employed as features by Gross et al. [21] and Bernal et al. [22] to determine the location of polyps.

In any case, these handcrafted-based methods can perform well, only for some ordinary polyps with fixed features. To further develop the semantic polyp segmentation, CNN-based architectures were adopted for the extraction of more discriminative characteristics. To improve the segmentation results for the colorectal polyps, Jha et al. [23] present the ResUNet++ architecture, a semantic segmentation neural network that employs residual and squeeze blocks, attention blocks, and atrous spatial pyramidal pooling (ASPP). Their architecture works effectively with a limited amount of images. The polyps in the CVC-ColonDB dataset were segmented by using a fully convolutional neural network (FCN-8S) by the authors of [24]. A method for image patch selection was applied in the training phase. Otsu thresholding is also used on the probability map as a postprocessing technique. They achieved a Dice score of 81%. Qadir et al. [25] used Mask R-CNN as a feature extractor to segment polyp areas in colonoscopy images by using different CNN architectures (ResNet50, ResNet101, and InceptionResNetV2). This method obtained a Dice score of 70.42% and a Jaccard index of 61.24% on CVC-ColonDB dataset. In their study [26], Poorneshwaran et al., utilized generative adversarial network (GAN) for the task of polyp image segmentation. The network achieved a Dice index of 88.48% on the CVC-ClinicDB dataset. Working also on colonoscopy images, Nguyen and Lee [27] employed a deep encoder–decoder technique. This work’s structure is made up of atrous convolution and depthwise separable convolution. On the CVC-ColonDB dataset, the suggested model received 88.9% of the Dice score and 89.35% of the Jaccard index.

On the other hand, several studies employed deep learning networks to segment digestive anomalies by using images collected from WCE the novel technology. Hajabdollahi et al. [28] suggest a simple and efficient approach for segmenting WCE bleeding images. MLP structure is used to classify appropriate colon channels. The authors proposed a quantized neural network with no multiplication which can be used as an automated diagnostic technique within the capsule. Ghosh et al. [29] used SegNet layers with three classes to train CNN. The training network is used to segment the endoscopic images, and the observed bleeding areas are indicated. Among several color planes, the hue saturation and value (HSV) color space provides the best performance. The framework achieved 94.42% global accuracy when tested on a publicly available dataset. The paper [30] presents a method for precise and efficient semantic calibration and refinement in convolutional neural networks (ConvNets) for real-time polyp segmentation from colonoscopy videos. The goal is to improve the accuracy of polyp segmentation, which is an important task in the diagnosis and monitoring of colon cancer. The method involves using ConvNets to analyze colonoscopy videos and identify polyps, and then refining the results by using a semantic calibration and refinement process. The authors in [31] present a new deep learning architecture for the simultaneous joint classification and segmentation of endoscopic images. The network consists of two parallel branches, a classification branch and a segmentation branch, which interact with each other through a synergistic interaction mechanism. The results show that the proposed architecture outperforms state-of-the-art methods on the benchmark datasets for endoscopic image classification and segmentation. The RA-UNet that was proposed by Jin et al. [32], focuses on preserving the high-level features of the image and enhances the resolution of the feature maps by using residual attention mechanisms in the encoding path. The AttU-Net part of the architecture focuses on refining the segmentation results by using an attention mechanism in the decoding path. The proposed RA-UNet architecture was tested on a public dataset of CT scans and showed improved accuracy and robustness compared to other state-of-the-art models for liver and tumor segmentation.

## 3. Materials and Methods

We present an overview of our suggested Attention Residual U-Net (AttResU-Net) approach in this section. We begin by providing a quick overview of the data preprocessing techniques used in this work. Then we describe the three model components: the residual encoding module, the attention gate (AG) module, and the residual decoding module. Finally, we describe how all of the elements are combined for further improvement. To improve the depth of the network while expediting network training and enhancing network performance, the usual convolution block in the encoder–decoder structure is replaced with a preactivated residual block. The AG module is included to record and screen for high-level features with additional spatial contextual information.

### 3.1. Data Preprocessing

The original datasets contain images of various resolutions. Some colonoscopic images have a dimension of 574 × 500, necessitating a much higher computing cost. As a result, the images must be resized before being fed to the segmentation models. We chose the appropriate size of 128 × 128 based on several experiments. Recent research has proved the benefits of data augmentation strategies in increasing the quality and size of training data to handle all data variations. Thus, various data augmentation techniques including vertical and horizontal flipping, rotating, and zooming are used in our case to extend the dataset and help in the successful accomplishment of the segmentation task. For most cases, pixel values of image data are integers with values ranging from 0 to 255. However, because the mechanism of neural networks uses minimal weight values, inputs with large values can slow the learning process. To that purpose, data normalization is considered to be a good solution, such that the value of each pixel should be between 0 and 1. That can be obtained by dividing all pixel values by 255, the largest pixel value. Normalizing the image can also make it more robust to changes in lighting conditions and other factors that might affect the overall brightness and contrast of the image.

### 3.2. AttResU-Net Architecture

We use a U-Net like structure for our network design, inspired by Ronneberger [33], with an encoder with numerous downsampling layers that extract features of various sizes and a decoder with several upsampling layers that scale the feature maps to the appropriate output resolution. To generate the skip connections, the intermediate outputs of the downsampling layers are merged with the intermediate outputs of their respective upsampling layers of the same size. Unlike Ronneberger work [33], we employ residual blocks rather than typical convolutional layers, as explained in Section 3.2.2. As described in Section 3.2.3, we also used attention modules to train the network to focus on relevant features for segmenting bleeding lesions and polyps.

#### 3.2.1. U-Net Architecture

U-Net is one of the most popular architectures used in image-segmentation tasks. This method was originally proposed by Ronneberger et al. [33] for biomedical image segmentation. Fundamentally, the U-Net is based on the fully convolutional network [34]. The UNet model is made up of two paths: contractive (encoder) and expansive (decoder). Deep features are learned along the first path based on the learned features. The encoder consists of a combination of convolution layers with filters of size 3 × 3 followed by ReLU and MaxPooling layers. The decoder, on the other hand, constructs an output segmentation map through a series of operations. This path comprises upconvolution, followed by convolution, ReLU, and MaxPooling layers. Through a skip connection, each encoder stage provides special features to the associated decoder stage. The key purpose of skip connection is to ensure that the model builds an increasingly exact output.

#### 3.2.2. Residual Units

**Figure 1:**
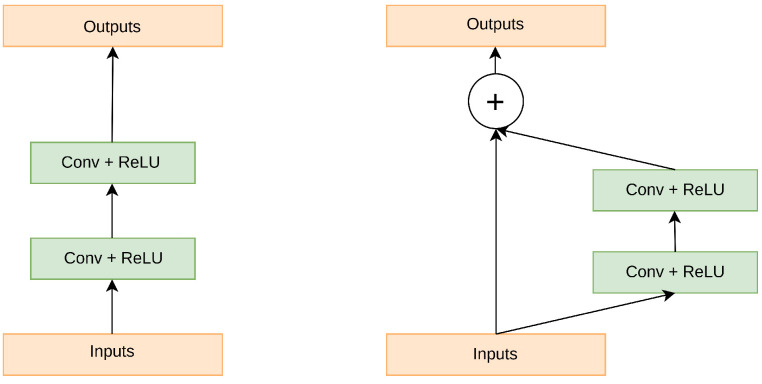
The difference between the ordinary convolutional block and the residual block.

Expanding network depth while training a deep neural network might potentially increase overall performance. However, simply stacking the CNN layer may impede the training process and result in exploding/vanishing gradients during backpropagation. Residual connections allow the gradient to bypass one or more layers in the neural network by adding the input of a layer to the output of a later layer. This allows the gradient to flow more directly to earlier layers, reducing the chance of the gradient becoming too small (vanishing) during backpropagation. Additionally, this structure allows the network to learn an identity mapping, allowing the network to learn an identity mapping when the layers are close to the identity function, making it easier to optimize [35]. A residual block is a building block used in deep residual neural networks and it mathematically defined as follows: Given an input tensor x, a residual block applies one or more layers of nonlinear transformations (such as convolutional layers, batch normalization, and ReLU activation) to produce an output tensor y. The final output of the residual block is then computed as the elementwise sum of the input tensor x and the output tensor y, added via an elementwise addition operation. This is represented mathematically in Equation (1) below,

(1) y=F(x)+x,

where F(x) is the nonlinear transformation applied to x. This structure allows the network to learn the residual mapping between the input x and the output y, allowing for the network to be much deeper and still be able to train effectively. This is because the residual block allows for the network to learn the difference between the input and output, rather than having to learn the entire function from scratch. This structure is also known as residual connection, which allows the gradients to flow more easily through the deep network, making it easier to train. Figure 1 depicts the residual coding structure.

#### 3.2.3. Attention Units

Deep learning’s attention mechanism may be linked back to the study of human vision. Humans communicate signals through neurons and brain processing after obtaining external visual information, which causes human attention to focus on the regions of interest and reduces the attention weight value of irrelevant regions. When the attention mechanism [36] is used in the deep learning process, the processing of human visual information is emulated by introducing the attention module. The attention to irrelevant areas will be limited by raising the information weight of the region of interest in the feature extraction process of the input image. Naive skip connections simply concatenate the encoder and decoder features, wasting computing resources and producing duplicate information. Because the encoder feature contains extensive location information, it is preferable to concentrate on relevant regions that are useful for determining the position of the item and determining the target structure of the object.

**Figure 2:**
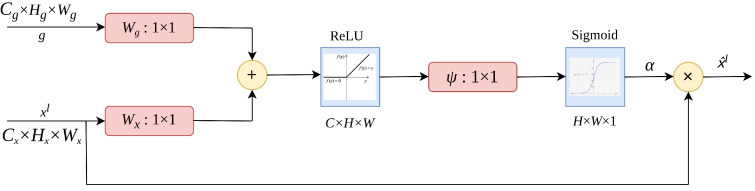
The structure of attention gate.

The attention gates as proposed by Oktay et al. [37] uses additive soft attention. An attention gate unit combines the input feature vector with the attention vector to produce a new, weighted feature vector. As depicted in Figure 2, the vectors $x^{l}$ and *g* are fed into the attention gate. The vector, *g*, is retrieved from the network’s next lowest layer. Because data originates from deeper in the network, the vector has lower dimensions and greater feature representation. Vector $x^{l}$ in the preceding case would be $C_{x}\times H_{x}\times W_{x}$ (filters × height × width), and vector *g* would be $C_{g}\times H_{g}\times W_{g}$. Vector $x^{l}$ undergoes a strided convolution, whereas vector *g* undergoes a 1 × 1 convolution. The two vectors are summed elementwise. As a result of this process, aligned weights get larger while unaligned weights become smaller. The resulting vector is subjected to a ReLU activation layer and a 1 × 1 convolution, which reduces the dimensions to H×W×1. This vector is sent via a sigmoid layer, which scales it between [0, 1], yielding the attention coefficients (weights), with coefficients closer to 1 indicating more significant information. Using trilinear interpolation, the attention coefficients are upsampled to the original dimensions of the $x^{l}$ vector. The attention coefficients α are multiplied by the original $x^{l}$ vector element by element (elementwise multiplication), scaling the vector ${\hat{x}}^{l}$ according to significance. This is then passed along normally in the skip connection.

#### 3.2.4. Our Network Structure

**Figure 3:**
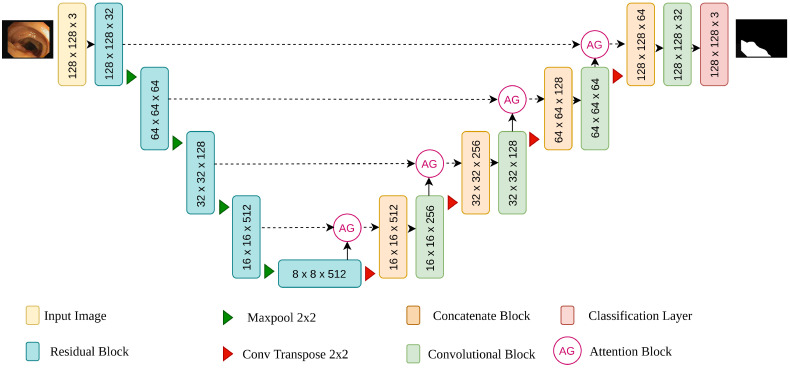
The proposed AttResU-Net framework.

**Figure 4:**
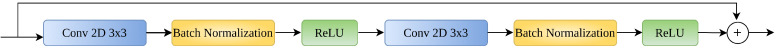
The proposed residual block structure.

In this study, we present a convolution neural network called attention residual UNet (AttResU-Net) for bleeding and polyps segmentation. The network adopts UNet as the network’s backbone, allowing a smooth translation from the image to the segmentation mask by combining the encoder and decoder structure with shallow semantic information. The network’s design may be separated into three components, as illustrated in Figure 3: encoder, skip connection with attention mechanism, and decoder. Different from the work of Liu et al. [38], the convolution block is replaced with an upgraded convolution residual module in the encoder and the decoder paths to prevent the gradient vanishing and explosion problem in the deep neural network and make the forward and backward propagation of information smoother. In addition to the suggested residual structure that is shown in Figure 4, this module contains a max pooling layer following it. The convolution kernel of the convolution operation is 3 × 3, and it is followed by a batch normalization layer and a ReLU. The max pooling layer reduces the size of the input image to half its original size. The number of input feature channels will be doubled after each encoder building block, and the image size will be half. The residual structure is not employed in the decoder path. The maximum pooling layer is replaced with an upsampling layer. Finally, we utilize the concatenation method to return the feature map to its original size. The upsampling rate is set to 2 in each decoder building block to guarantee that the output is the same size as the encoder output. To make better use of the information in the multiresolution feature map, the attention gate is embedded in the skip connection part to strengthen the useful features in the channel dimension and suppress the inaccurate background features, hence improving the computational efficiency of the network model. The output of this part is then concatenated with the output of the previous layer of the decoder and fed into the current layer’s decoder.

### 3.3. SegNet Architecture

The SegNet concept was suggested for the first time by Badrinarayanan et al. [39] in 2017. SegNet is a deep neural network designed to simulate scene parts, similar to a road picture segmentation tool. It uses around ten times fewer learning parameters than the UNet model. The encoder–decoder architecture, as in VGG16, comprises 13 convolutional layers with filters of size 3 × 3, batch normalization, ReLU, and five layers of 2 × 2 max-pooling. A decoder is a reflected version of the encoder, it generates the segmentation mask by using pooling indices from the max-pooling layers of the associated encoder. Toward the end, there is a softmax activation that produces a classified binary image that divides pixels into two categories (abnormality/background).

### 3.4. V-Net Architecture

In medical image segmentation, V-Net [40] is a well-known 3D fully convolutional neural network. It is symmetrically constructed and consists of an encoder and a decoder. The encoder extracts relevant features from the input image, and the decoder reconstructs those features to produce the final segmentation results. The network’s left side is a compression path, whereas the network’s right side decompresses the signal until it reaches its original size. The network’s left side is subdivided into stages that perform at various resolutions. Each one of those stages has one to three convolutional layers. At every stage, a residual function is learned. To train a residual function, the input of each stage is used in the convolutional layers, processed via the nonlinearities, and added to the output of the last convolutional layer of that stage. Convolutions are done in each step by using volumetric kernels with a size 5 × 5 × 5. Convolution with 2 × 2 × 2 kernels performed with stride 2 reduces resolution throughout the compression path.

## 4. Results

Our approach aims for GI tract abnormalities (bleeding and polyps) semantic segmentation, in order to generate pixelwise segmentation masks for each endoscopic and colonoscopic image. In this section, we will initially describe the three used datasets. Then, we will introduce the evaluation metrics and the experimental setup. Finally, both quantitative and qualitative findings will be discussed.

### 4.1. Datasets

The suggested approach was validated by using three publicly available datasets. The first dataset has labeled red lesions in WCE images, whereas the second is the Kvasir-SEG dataset, and the CVC-ClinicDB dataset, which comprises colonoscopic images. These three datasets are briefly discussed below.

#### 4.1.1. Red Lesion Dataset

The publicly available WCE dataset for small bowel lesions has been obtained from [41]. It contains 1131 bleeding lesions and 2164 normal images. All frames were manually annotated by expert physicians. The images have 320 × 320 or 512 × 512 resolutions. The dataset was created with images from various cameras, such as MiroCam, PillCam SB1, SB2, and SB3, and with various red lesions, such as angioectasias, angiodysplasias, hemorrhage, and others.

#### 4.1.2. Kvasir-SEG

The Kvasir-SEG was first introduced by Jha et al., in [42]. It consists of 1000 polyp images acquired by high-resolution electromagnetic imaging systems. It also contains the 1000 corresponding ground truth masks that were annotated by expert endoscopists from Oslo University Hospital (Norway). The dataset includes images with resolutions ranging from 332 × 487 to 1920 × 1072 pixels. The images contain polyps of different sizes (large, medium, and small). All images are encoded using JPEG compression.

#### 4.1.3. CVC-ClinicDB Dataset

**Figure 5:**
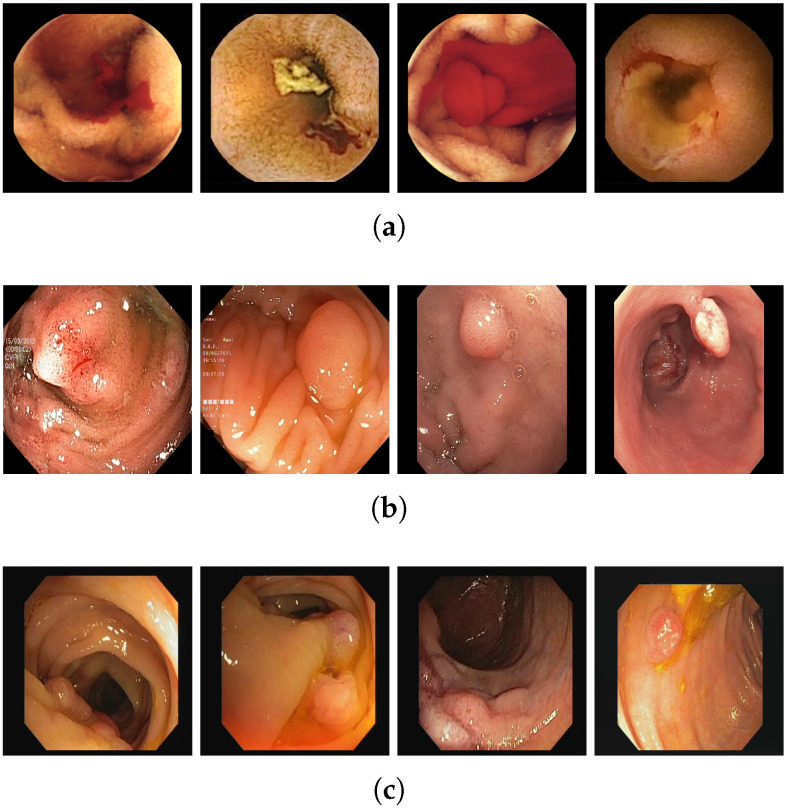
Example frames from the three publicly available datasets (**a**) MICCAI2017 (**b**) Kvasir-SEG (**c**) CVC-ClinicDB.

The third dataset was first presented in 2012 by Bernal et al., in [22]. It is an open-access dataset of 612 colonoscopy images of size 574 × 500 pixels. Each pixel in the images is annotated as polyp or background. The frames are obtained from 31 polyp video sequences collected from 23 patients. Table 1 depicts some of the three datasets details, and Figure 5 shows some of the bleeding and polyp images from different datasets.

### 4.2. Implementation Details

During the segmentation process, all the experiments were implemented in Python 3.6 by using the Keras framework with Tensorflow as a backend. They were conducted on an NVIDIA GeForce GTX 1050 (4 GB GPU memory) Core i7. We worked with a Ubuntu 16.04 LTS operating system with Cuda 8.0.61 version installed. For this study, we randomly selected 60% of each dataset for the training, 20% for the validation, and the resting 20% for the test set. We have manually conducted different experiments with multiple sets of hyperparameters on the same dataset and with the same model in order to get the optimal set of hyperparameters. Those sets were chosen based on the empirical evaluation. In the training phase of all the cases, our models performed well with the batch size of 40, Adam as an optimizer, and 50 for the epochs. $\beta_{1}$ and $\beta_{2}$ were set to 0.9 and 0.99, respectively. In addition, to prevent overfitting, we employed an early stopping mechanism. Early stopping is a type of regularization used when training a model by using an iterative approach like gradient descent. Because all neural networks train utilizing gradient descent, early stopping is a strategy that can be applied to any task. With each iteration, this strategy improves the model fit to the training data. This increases the model performance on data from the test set to a point. However, enhancing the model’s fit to the training data after that point leads to higher generalization error. Early stopping criteria specify how many iterations may be performed before the model begins to overfit.

### 4.3. Evaluation Metrics

There are several metrics for assessing and comparing the models’ performance. For medical image-segmentation applications, the most often utilized metrics are the Dice coefficient, Jaccard index, also known as intersection over union (IoU), and accuracy. These metrics are calculated as follows.

Dice Coefficient: The Dice coefficient is a relative metric for comparing the pixelwise results between given ground truth and predicted segmentation. Its value range is from 0 to 1, with 0 indicating no spatial overlap between two sets of binary segmentation results and 1 representing total overlap. The Dice coefficient is equal to twice the number of elements shared by both sets A and B, divided by the total number of elements in each set.

(2) $$\mathrm{Dice}\;\mathrm{Coefficient}\;\left(A,B\right)\;=\frac{2\times |A\cap B|}{\left|A|+|B\right|}$$

Jaccard Index: Another typical measure for evaluating a segmentation approach is the Jaccard index. It computes the similarity between predicted (A) and ground truth (B), as illustrated in the equation below:

(3) $$\mathrm{Jaccard}\;\mathrm{Index}\;\left(A,B\right)\;=\frac{\left|A\cap B\right|}{A\cup B}=\frac{\left|A\cap B\right|}{\left|A|+|B|+|A\cap B\right|}$$

Accuracy: A high accuracy indicates that the pixels were correctly classified,

(4) $$\mathrm{Accuracy}=\frac{\mathrm{TP}\;+\;\mathrm{TN}}{\mathrm{TP}\;+\;\mathrm{FP}\;+\;\mathrm{FN}\;+\;\mathrm{TN}},$$

where (TP), (TN), (FP), and (FN) represent true positive, true negative, false positive, and false negative, respectively.

### 4.4. Experimental Results

To further verify the effectiveness of our method, we trained UNet, SegNet, V-Net, AttU-Net, and AttResU-Net on the MICCAI 2017, Kvasir-SEG, and CVC-ClinicDB datasets and tested them on the test sets. The accuracy, Dice coefficient, and intersection over union (IoU) of each model were calculated. The test results of each model on the three different datasets are presented in this subsection. In our experiments, we choose 80% of the images for training and the rest images are used for testing. We compare AttResU-Net with its baseline of U-Net, as well as two recently proposed networks including SegNet and V-Net.

#### 4.4.1. Results on MICCAI 2017

**Figure 6:**
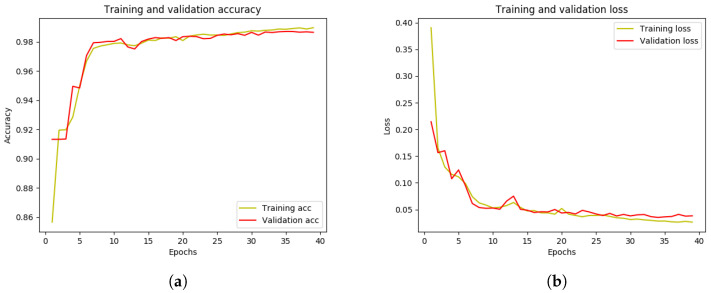
The learning and loss curve of the AttResU-Net model on MICCAI 2017 dataset.

Table 2 gives the performances of our proposed AttResU-Net and compared different SOTA algorithms on the test set of MICCAI 2017. It can be seen that the proposed AttResU-Net achieves the best performance in three metrics. As given in Table 2, AttResU-Net achieves an accuracy of 99.16%, a dice coefficient of 94.91%, and a Jaccard index of 90.32% on the MICCAI 2017 datasets, outperforming the original U-Net by 6.42%, 8.37%, and 12.35% gains, respectively. We can clearly notice that AttResU-Net defeats the U-Net network, which has the best outcome compared with the two other segmentation models V-Net and SegNet. It also outperforms AttU-Net presented by Oktay et al. [37] as an attention gate (AG) module based on the structure of the U-Net. To create the attention weight maps, the AG module uses the feature maps from the encoder as the input signal and the related upsampling results as the gating signal. They then multiplied the weighted feature maps by the original feature maps and replaced the U-Net skip connections with the weighted feature maps. The target regions were more responsive to the weighted feature maps. This can be explained by the importance of the two powerful modules that we add to the traditional U-Net, including the residual block and attention mechanism. On the other hand, the running time of our proposed framework takes more than five times compared to U-Net. We can say that this is very normal given the complexity of our network. However, as it is well known in the medical field, the precision of the results is favored over the running time. To further show the training process of our model, we present learning and loss curves in Figure 6. The first figure shows the accuracy curve of the segmentation model. The x-axis represents the number of training epochs and the y-axis represents the accuracy of the model. As the model trains, the accuracy increases from an initial value of approximately 91% to a final value of approximately 99%. This increase in accuracy indicates that the model is improving and learning to make more accurate predictions. It can also be seen that the accuracy levels off after approximately 40 epochs, which suggests that the model has reached a convergence point and is no longer improving. As such, we can consider 40 epochs to be the optimal number of training epochs for this model. The final accuracy of 99% is relatively high, indicating that the model is making accurate predictions most of the time. The second figure shows a loss curve of our segmentation model. The loss curve is a graph that represents the change in the loss function over time as the model trains. As the model trains, the loss decreases from an initial value of around 0.22 to a final value of around 0.04. This decrease in loss indicates that the model is improving and learning to make more accurate predictions. The final loss of 0.04 is relatively low, indicating that the model is making accurate predictions.

**Figure 7:**
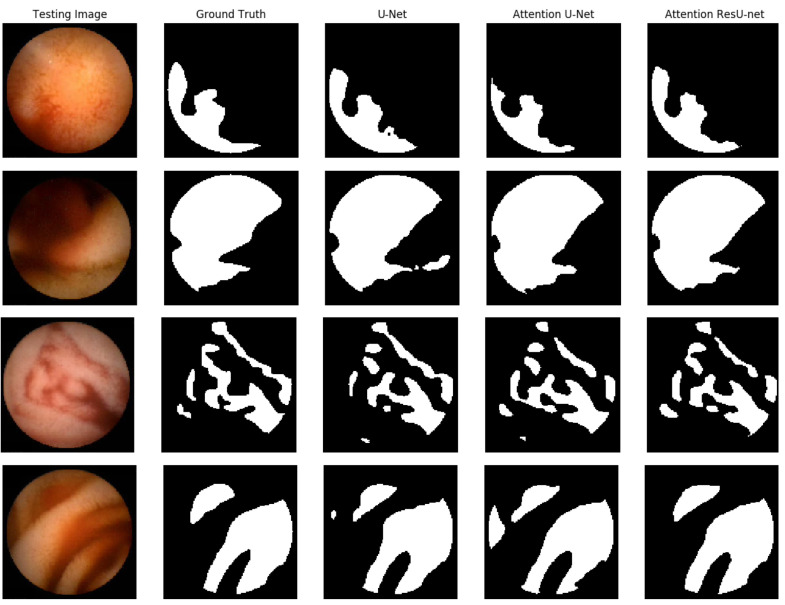
Qualitative results comparison on the MICCAI 2017 dataset.

In Figure 7, we demonstrate some qualitative endoscopic lesion segmentation findings from the MICCAI 2017 test set acquired by using several approaches. The four original images show varied lesions in regard to appearance and size, and there are no apparent structural borders between normal and lesion areas. Furthermore, certain areas with poor quality make data analysis difficult. All of these present challenges in distinguishing aberrant from normal areas. However, we can observe that the segmentation of our suggested AttResU-Net is close to the ground truth. On the contrary, other methods can capture big abnormal regions successfully but may miss certain tiny abnormal regions.

#### 4.4.2. Results Kvasir-SEG

Table 3 displays the results of AttResU-Net, AttU-Net, UNet, SegNet, and V-Net. The suggested model achieved the maximum Dice coefficient, mIoU, and accuracy for the Kvasir-SEG dataset, as shown in Table 3. AttU-Net attained a high level of accuracy. The Dice coefficient and mIoU scores, on the other hand, are not competitive, which are essential metrics for semantic segmentation tasks. In terms of mIoU, the suggested design outperformed the baseline architectures by a large margin.

**Figure 8:**
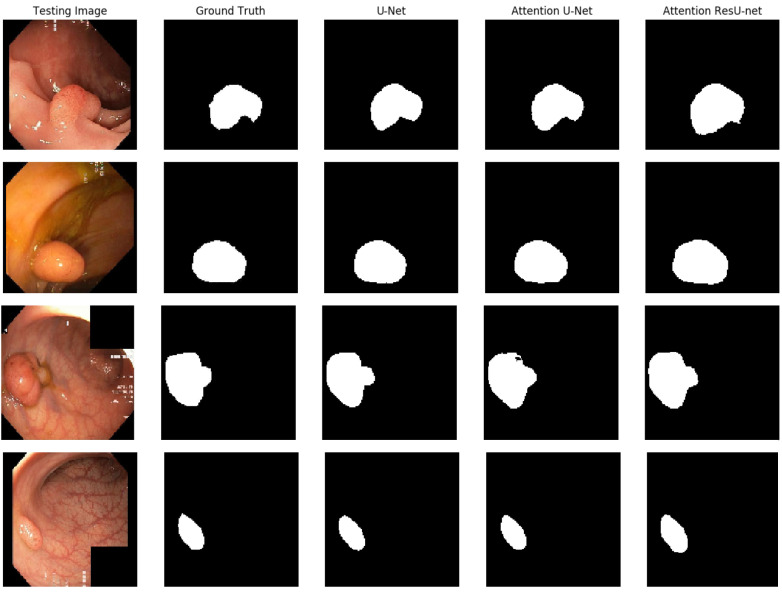
Qualitative results comparison on the Kvasir-SEG dataset.

The qualitative outcome for the polyp detection and localization task is shown in Figure 8. It is clear that all of the algorithms can detect polyps and provide high-quality masks. The best segmentation results are achieved for AttU-Net and AttResU-Net, especially for minor details, as seen in the first example. On basically all samples, our suggested network yields comparatively better predictions.

#### 4.4.3. Results CVC-ClinicDB

**Figure 9:**
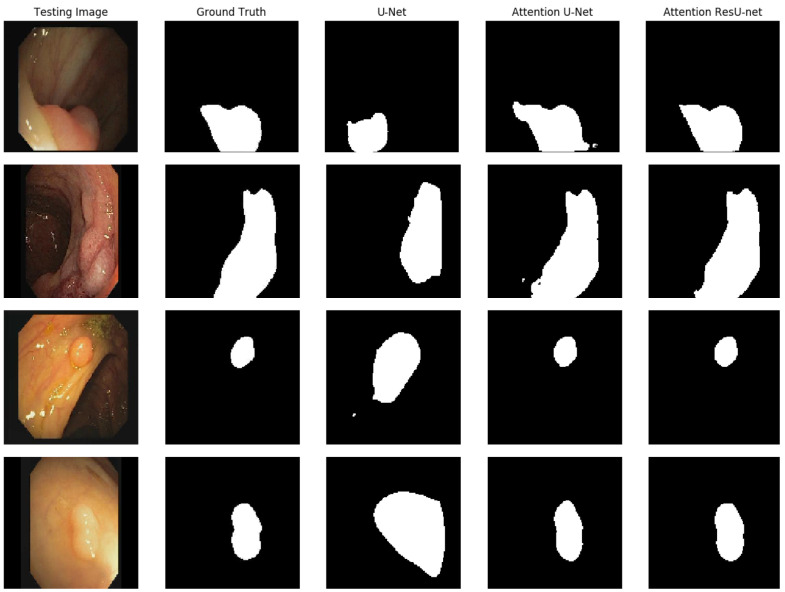
Qualitative results comparison on the CVC-ClinicDB dataset.

To make segmentation more challenging, we tested our model by using the CVC-ClinicDB dataset, which only comprises 612 clinical digital images. We ran additional tests to conduct an in-depth performance investigation of autonomous polyp segmentation. As a result, we tried model generalization to test the suggested architecture’s generalizability on a new dataset. Generalizability would be a significant step toward developing a clinically appropriate model. Table 4 displays the outcomes for all models on the CVC-ClinicDB dataset. The suggested model was shown to have the highest Dice coefficient, mIoU, and accuracy. The qualitative findings for all models are shown in Figure 9. The suggested model may attain comparable patterns to those of the ground truth, with some exceptions for the predictions. The advantage of AttResU-Net over the baseline is demonstrated by Table 4 and Figure 9.

## 5. Discussion

### 5.1. Comparison with State-of-the-Art Studies

To better evaluate the performance of our models, we compare them with the state-of-the-art methods on every single dataset. As shown in Table 5 AttResU-Net outperformed the existing studies conducted on the WCE red lesion dataset in terms of accuracy with a difference of 10.16%. Table 6 compares the suggested approaches’ performance to that of [30,43]. These existing networks are based on deep learning techniques such as ResUNet, PSP-Net, Attention-UNet, and CE-Net. ResNet-mod has the highest Dice coefficient which is 79.09%, whereas CE-Net has the best mIoU (67.28%). The advantage of our model is having a high Dice score and a high IoU at the same time contrary to ResNet-mod which has a low mIoU (just 43.64%). In the Table 7, the comparison of CVC-ClinicDB is depicted. We can see that ResNet50 achieved the best Dice score (71.6%) and the best IoU 63.9%) with a big difference compared to our Dice coefficient (74.39%).

### 5.2. Limitations

The suggested architecture yields good results on the three different used datasets, even if we are dealing with different modalities (colonoscopy/endoscopy), different types of abnormalities (red lesions/polyps) different sizes of lesions (large, medium, tiny), and different shapes and textures. The good performance of the proposed models can be clearly noticed from both the qualitative and quantitative results. However, U-Net also provided competitive segmentation findings. Based on our empirical evaluation, we optimized our implementation as much possible by selecting the optimal hyperparameters. However, further optimization can occur. One of the weaknesses of our architecture is that it is computationally expensive compared to other image-segmentation models. This is due to the complexity of the U-Net architecture, as well as the additional computational overhead of the attention mechanism. This can make it difficult to use the AttResU-Net in real-time applications, or in situations in which computational resources are limited. Moreover, AttResU-Net may struggle with images that contain large variations in intensity or texture. The attention mechanism used in the AttResU-Net is designed to focus on the most important regions of an image, but it may not always be effective in these cases, leading to suboptimal performance. Finally, the performance of the AttResU-Net can also be influenced by the choice of loss function and other hyperparameters. Fine tuning these parameters is important for achieving optimal results, but it can be time consuming and require a significant amount of experimentation.

### 5.3. Future Work

To overcome the points of weakness mentioned above, we plan to work on different strategies in the future works in order to improve the overall performance of our system. There are several ways that we suggest to improve the AttResU-Net architecture.

- Increasing the depth of the network: This could improve the network’s ability to capture complex features and relationships between the inputs and outputs.
- Adding skip connections: This could help improve the gradient flow and reduce the problem of vanishing gradients.
- Ensemble learning: Training multiple models and combining their outputs could potentially lead to improved results.
- Hyperparameter tuning: Fine tuning the model’s hyperparameters, such as the learning rate, batch size, and weight initialization method, could potentially lead to improved performance.
- Data augmentation: Adding data augmentation techniques, such as random rotations, flips, or shifts, could help the network generalize better to new data.
- Transfer learning: Using a pretrained model as the starting point for the AttResU-Net architecture could potentially improve the results.
- Modifying the attention mechanism: Experimenting with different attention mechanisms, such as self-attention or multihead attention, could lead to improved performance.

## 6. Conclusions

In this study, we introduced a novel AttResU-Net model for the polyp and bleeding segmentation challenge, which integrated an attention mechanism and residual units into the traditional U-Net architecture to increase semantic segmentation performance. AttResU-Net adaptively rescaled features by adding a sequence of attention units among matching downsampling and upsampling processes, enhancing local responses of downsampling residual features as well as the recovery effects of the upsampling process. Experiment findings on three endoscopic and colonoscopic datasets benchmarks revealed that AttResU-Net exceeded U-Net by a wide margin and achieved better performance compared to other predominant polyp and bleeding segmentation algorithms. Nevertheless, because of computational resource constraints, our model used 128 × 128 as input size to create the segmentation network, resulting in additional context information loss. As a consequence, in the future, we want to extend our AResU-Net to a 3D network with a bigger size input image in order to get better segmentation results and apply it to additional medical image-segmentation jobs for further testing. In addition, several more strong feature-extraction modules will be investigated in order to get an additional performance boost.

## Figures and Tables

**Table 1:** Distribution of the datasets used in our study.

Dataset	Images	Train/Test	Imaging Type
Red Lesion in VCE	3295	2796/652	WCE
Kvasir-SEG	1000	800/200	Colonoscopy
CVC-ClinicDB	612	490/122	Colonoscopy

**Table 2:** Quantitative results on MICCAI 2017 dataset.

Architecture	Mean Accuracy (%)	Dice Coefficient (%)	Mean IoU (%)
SegNet	78.14	83.13	75.31
V-Net	87.66	91.64	66.71
ResAttU-Net [38]	92.50	82.72	70.93
U-Net	92.74	86.54	77.97
AttU-Net [37]	94.14	89.19	80.62
AttResU-Net	99.16	94.91	90.32

**Table 3:** Quantitative results on Kvasir-SEG dataset.

Architecture	Mean Accuracy (%)	Dice Coefficient (%)	Mean IoU (%)
SegNet	89.62	61.68	50.47
V-Net	92.56	76.03	63.38
U-Net	93.37	78.03	67.91
AttU-Net [37]	93.74	80.90	68.18
AttResU-Net	97.55	91.76	84.78

**Table 4:** Quantitative results on CVC-ClinicDB dataset.

Architecture	Mean Accuracy (%)	Dice Coefficient (%)	Mean IoU (%)
SegNet	91.69	53.04	51.31
V-Net (2D)	94.32	65.76	87.10
U-Net	95.15	74.39	61.84
AttU-Net [37]	97.46	90.85	83.26
AttResU-Net	97.58	91.85	84.95

**Table 5:** Comparison of bleeding segmentation approaches on the WCE dataset.

Author	Method	Accuracy
Figueiredo et al. [44]	Computerized method	86.9%
Liangpunsakul et al. [45]	Suspected blood indicator tool	73%
Sainju et al. [46]	Statistical features and region growing	87%
Our proposed method	Attention residual U-net	99.16%

**Table 6:** Quantitave comparison of the segmentation results with other works on the Ksavir-SEG dataset.

Author	Method	Dice Coefficient (%)	Mean IoU (%)
Jha et al. [43]	ResUNet	51.44	43.64
	ResUnet-mod	79.09	42.87
Wu et al. [30]	PSP-Net	75.92	66.27
	Attention U-Net	75.46	65.93
	ResUNet++	76.12	66.42
	CE-Net	76.97	67.28
Our proposed method	Attention residual U-net	91.76	84.78

**Table 7:** Segmentation comparison of our best-performing model with other studies on the CVC-ClinicDB dataset.

Author	Method	Dice Coefficient (%)	Mean IoU (%)
Qadir et al. [25]	Resnet 50	71.60	63.90
	Resnet101	70.42	63.30
	Resnet Inception	70.31	63.60
Jha et al. [43]	ResUNet	45.10	45.70
Our proposed method	Attention Residual U-Net	91.85	84.95

## Data Availability

Publicly available datasets were analyzed in this study. The CVCClinicDB datasets are publicly available here: https://polyp.grandchallenge.org/CVCClinicDB/ (accessed on 15 September 2022). The Kvasir-Seg dataset is publicly available here: https://datasets.simula.no/kvasir-seg/ (accessed on 15 September 2022).

## References

[1] Ramamurthy K., George T.T., Shah Y., Sasidhar P. (2022). A Novel Multi-Feature Fusion Method for Classification of Gastrointestinal Diseases Using Endoscopy Images. Diagnostics.

[2] Mahmood S., Fareed M.M.S., Ahmed G., Dawood F., Zikria S., Mostafa A., Jilani S.F., Asad M., Aslam M. (2022). A Robust Deep Model for Classification of Peptic Ulcer and Other Digestive Tract Disorders Using Endoscopic Images. Biomedicines.

[3] Ozyoruk K.B., Gokceler G.I., Bobrow T.L., Coskun G., Incetan K., Almalioglu Y., Mahmood F., Curto E., Perdigoto L., Oliveira M. (2021). EndoSLAM dataset and an unsupervised monocular visual odometry and depth estimation approach for endoscopic videos. Med. Image Anal..

[4] Stewart B.J., Ferdinand J.R., Clatworthy M.R. (2020). Using single-cell technologies to map the human immune system—Implications for nephrology. Nat. Rev. Nephrol..

[5] Hong T.C., Liou J.M., Yeh C.C., Yen H.H., Wu M.S., Lai I.R., Chen C.C. (2020). Endoscopic submucosal dissection comparing with surgical resection in patients with early gastric cancer–a single center experience in Taiwan. J. Formos. Med. Assoc..

[6] Charfi S., Ansari M.E. (2018). Computer-aided diagnosis system for colon abnormalities detection in wireless capsule endoscopy images. Multimed. Tools Appl..

[7] Souaidi M., El Ansari M. (2019). Multi-scale analysis of ulcer disease detection from WCE images. IET Image Process..

[8] Xie L., Cai W., Gao Y. (2022). DMCGNet: A Novel Network for Medical Image Segmentation With Dense Self-Mimic and Channel Grouping Mechanism. IEEE J. Biomed. Health Inform..

[9] Yu Z., Chen J., Liu Y., Chen Y., Wang T., Nowak R., Lv Z. (2022). DDCNN: A Deep Learning Model for AF Detection from a Single-Lead Short ECG Signal. IEEE J. Biomed. Health Inform..

[10] Liu M., Hu L., Tang Y., Wang C., He Y., Zeng C., Lin K., He Z., Huo W. (2022). A deep learning method for breast cancer classification in the pathology images. IEEE J. Biomed. Health Inform..

[11] Sriker D., Cohen D., Cahan N., Greenspan H. Improved segmentation by adversarial u-net. Proceedings of the Medical Imaging 2021: Computer-Aided Diagnosis. International Society for Optics and Photonics.

[12] Lafraxo S., El Ansari M. (2020). GastroNet: Abnormalities Recognition in Gastrointestinal Tract through Endoscopic Imagery using Deep Learning Techniques. Proceedings of the 2020 8th International Conference on Wireless Networks and Mobile Communications (WINCOM).

[13] Feng R., Lei B., Wang W., Chen T., Chen J., Chen D.Z., Wu J. (2020). SSN: A stair-shape network for real-time polyp segmentation in colonoscopy images. Proceedings of the 2020 IEEE 17th International Symposium on Biomedical Imaging (ISBI).

[14] Garbaz A., Lafraxo S., Charfi S., El Ansari M., Koutti L. (2022). Bleeding classification in Wireless Capsule Endoscopy Images based on Inception-ResNet-V2 and CNNs. Proceedings of the 2022 IEEE Conference on Computational Intelligence in Bioinformatics and Computational Biology (CIBCB).

[15] Oukdach Y., Kerkaou Z., El Ansari M., Koutti L., El Ouafdi A.F. (2022). Gastrointestinal diseases classification based on deep learning and transfer learning mechanism. Proceedings of the 2022 9th International Conference on Wireless Networks and Mobile Communications (WINCOM).

[16] Souaidi M., El Ansari M. (2022). Multi-Scale Hybrid Network for Polyp Detection in Wireless Capsule Endoscopy and Colonoscopy Images. Diagnostics.

[17] Hwang S., Celebi M.E. (2010). Polyp detection in wireless capsule endoscopy videos based on image segmentation and geometric feature. Proceedings of the 2010 IEEE International Conference on Acoustics, Speech and Signal Processing.

[18] Fu Y., Zhang W., Mandal M., Meng M.Q.H. (2013). Computer-aided bleeding detection in WCE video. IEEE J. Biomed. Health Inform..

[19] Ganz M., Yang X., Slabaugh G. (2012). Automatic segmentation of polyps in colonoscopic narrow-band imaging data. IEEE Trans. Biomed. Eng..

[20] Mamonov A.V., Figueiredo I.N., Figueiredo P.N., Tsai Y.H.R. (2014). Automated polyp detection in colon capsule endoscopy. IEEE Trans. Med. Imaging.

[21] Gross S., Kennel M., Stehle T., Wulff J., Tischendorf J., Trautwein C., Aach T. (2009). Polyp segmentation in NBI colonoscopy. Bildverarbeitung für die Medizin 2009.

[22] Bernal J., Sánchez J., Vilarino F. (2012). Towards automatic polyp detection with a polyp appearance model. Pattern Recognit..

[23] Jha D., Smedsrud P.H., Riegler M.A., Johansen D., De Lange T., Halvorsen P., Johansen H.D. (2019). Resunet++: An advanced architecture for medical image segmentation. Proceedings of the 2019 IEEE International Symposium on Multimedia (ISM).

[24] Akbari M., Mohrekesh M., Nasr-Esfahani E., Soroushmehr S.R., Karimi N., Samavi S., Najarian K. (2018). Polyp segmentation in colonoscopy images using fully convolutional network. Proceedings of the 2018 40th Annual International Conference of the IEEE Engineering in Medicine and Biology Society (EMBC).

[25] Qadir H.A., Shin Y., Solhusvik J., Bergsland J., Aabakken L., Balasingham I. (2019). Polyp detection and segmentation using mask R-CNN: Does a deeper feature extractor CNN always perform better?. Proceedings of the 2019 13th International Symposium on Medical Information and Communication Technology (ISMICT).

[26] Poorneshwaran J., Kumar S.S., Ram K., Joseph J., Sivaprakasam M. (2019). Polyp segmentation using generative adversarial network. Proceedings of the 2019 41st Annual International Conference of the IEEE Engineering in Medicine and Biology Society (EMBC).

[27] Nguyen Q., Lee S.W. (2018). Colorectal segmentation using multiple encoder-decoder network in colonoscopy images. Proceedings of the 2018 IEEE First International Conference on Artificial Intelligence and Knowledge Engineering (AIKE).

[28] Hajabdollahi M., Esfandiarpoor R., Soroushmehr S., Karimi N., Samavi S., Najarian K. (2018). Segmentation of bleeding regions in wireless capsule endoscopy images an approach for inside capsule video summarization. arXiv.

[29] Ghosh T., Li L., Chakareski J. (2018). Effective deep learning for semantic segmentation based bleeding zone detection in capsule endoscopy images. Proceedings of the 2018 25th IEEE International Conference on Image Processing (ICIP).

[30] Wu H., Zhong J., Wang W., Wen Z., Qin J. Precise Yet Efficient Semantic Calibration and Refinement in ConvNets for Real-time Polyp Segmentation from Colonoscopy Videos. Proceedings of the AAAI Conference on Artificial Intelligence.

[31] Zhu M., Chen Z., Yuan Y. (2021). DSI-net: Deep synergistic interaction network for joint classification and segmentation with endoscope images. IEEE Trans. Med. Imaging.

[32] Jin Q., Meng Z., Sun C., Cui H., Su R. (2020). RA-UNet: A hybrid deep attention-aware network to extract liver and tumor in CT scans. Front. Bioeng. Biotechnol..

[33] Ronneberger O., Fischer P., Brox T. (2015). U-net: Convolutional networks for biomedical image segmentation. Proceedings of the International Conference on Medical Image Computing and Computer-Assisted Intervention.

[34] Long J., Shelhamer E., Darrell T. Fully convolutional networks for semantic segmentation. Proceedings of the IEEE Conference on Computer Vision and Pattern Recognition.

[35] He K., Zhang X., Ren S., Sun J. Deep residual learning for image recognition. Proceedings of the IEEE Conference on Computer Vision and Pattern Recognition.

[36] Vaswani A., Shazeer N., Parmar N., Uszkoreit J., Jones L., Gomez A.N., Kaiser Ł., Polosukhin I. (2017). Attention is all you need. Adv. Neural Inf. Process. Syst..

[37] Oktay O., Schlemper J., Folgoc L.L., Lee M., Heinrich M., Misawa K., Mori K., McDonagh S., Hammerla N.Y., Kainz B. (2018). Attention u-net: Learning where to look for the pancreas. arXiv.

[38] Liu Y.C., Tan D.S., Chen J.C., Cheng W.H., Hua K.L. (2019). Segmenting hepatic lesions using residual attention U-Net with an adaptive weighted dice loss. Proceedings of the 2019 IEEE International Conference on Image Processing (ICIP).

[39] Badrinarayanan V., Kendall A., Cipolla R. (2017). Segnet: A deep convolutional encoder-decoder architecture for image segmentation. IEEE Trans. Pattern Anal. Mach. Intell..

[40] Milletari F., Navab N., Ahmadi S.A. (2016). V-net: Fully convolutional neural networks for volumetric medical image segmentation. Proceedings of the 2016 Fourth International Conference on 3D Vision (3DV).

[41] Coelho P., Pereira A., Salgado M., Cunha A. (2018). A deep learning approach for red lesions detection in video capsule endoscopies. Proceedings of the International Conference Image Analysis and Recognition.

[42] Jha D., Smedsrud P.H., Riegler M.A., Halvorsen P., Lange T.D., Johansen D., Johansen H.D. (2020). Kvasir-seg: A segmented polyp dataset. Proceedings of the International Conference on Multimedia Modeling.

[43] Jha D., Smedsrud P.H., Johansen D., de Lange T., Johansen H.D., Halvorsen P., Riegler M.A. (2021). A comprehensive study on colorectal polyp segmentation with ResUNet++, conditional random field and test-time augmentation. IEEE J. Biomed. Health Inform..

[44] Figueiredo I.N., Kumar S., Leal C., Figueiredo P.N. (2013). Computer-assisted bleeding detection in wireless capsule endoscopy images. Comput. Methods Biomech. Biomed. Eng. Imaging Vis..

[45] Liangpunsakul S., Mays L., Rex D.K. (2003). Performance of Given suspected blood indicator. Am. J. Gastroenterol..

[46] Sainju S., Bui F.M., Wahid K.A. (2014). Automated bleeding detection in capsule endoscopy videos using statistical features and region growing. J. Med. Syst..

